# Towards greener intravitreal injections: the Oxford Eye Hospital experience

**DOI:** 10.1038/s41433-025-03899-8

**Published:** 2025-07-09

**Authors:** Ariel Y. Ong, Thomas M. W. Buckley, Johannes Birtel, Samantha R. de Silva, Niamh Stone, Peter Charbel Issa

**Affiliations:** 1 Oxford Eye Hospital, Oxford University Hospitals NHS Foundation Trust, Oxford, UK; 2 Institute of Ophthalmology, University College London, London, UK; 3 Department of Ophthalmology, University Medical Center Hamburg-Eppendorf, Hamburg, Germany; 4 Nuffield Laboratory of Ophthalmology, Nuffield Department of Clinical Neurosciences, University of Oxford, Oxford, UK; 5 Technical University of Munich, School of Medicine and Health, Department of Ophthalmology, TUM University Hospital, Munich, Germany

**Keywords:** Retinal diseases, Health services

The imperative for sustainability in healthcare continues to grow. Intravitreal injections (IVIs) have become a cornerstone of eye care services, and are increasing in number year on year [1]. This therefore represents a key area where environmentally conscious practices can and should be implemented. While carbon emissions relating to infrastructure and travel have been a key focus of sustainability efforts, addressing procedural waste from IVIs represents a “low hanging fruit” that warrants equal attention [2].

At the Oxford Eye Hospital (OEH), we have deployed strategies to reduce the environmental footprint of IVI services by addressing both travel-related emissions and procedural practices. OEH serves a population of over 800,000 people living in Oxfordshire, and covers a geographical area spanning approximately 2600 km^2^. This comprises one tertiary centre and an outreach clinic in a peripheral hospital with enhanced imaging services, to facilitate virtual reviews and increased capacity for IVI clinics while reducing patient travel. Same-day bilateral IVIs are offered where indicated.

We have implemented stepwise changes in the standard operating procedure (SOP) for IVIs at OEH over the last eight years (Fig. 1A), representing a gradual move towards a sustainable approach to performing IVIs which was always backed by current literature and proved to be safe [2]. This includes cessation of postoperative topical antibiotics and periocular skin cleaning with povidone iodine, the optional omission of a surgical drape and lid speculum, and the use of non-sterile gloves in place of sterile gloves. Intravitreal packs are no longer in routine use.

**Fig. 1:**
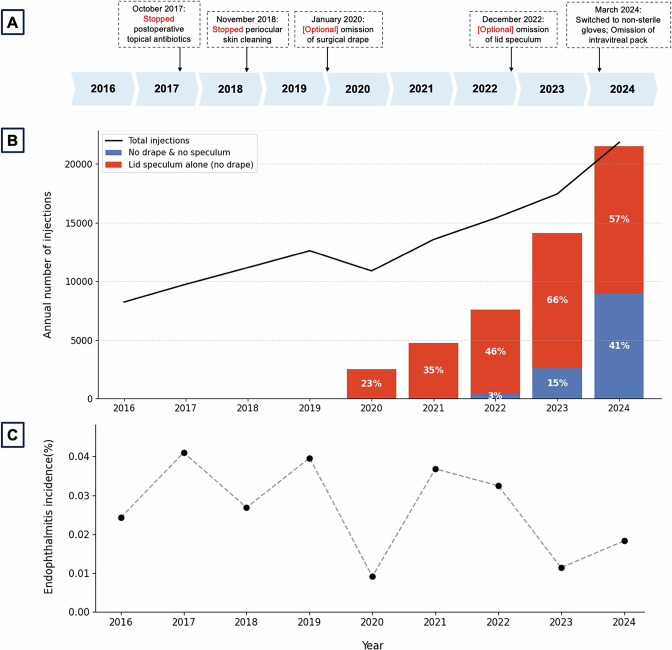
Evolution of intravitreal injection standard operating protocol, uptake of proposed changes, and safety outcomes at the Oxford Eye Hospital from 2016–2024. **A** Stepwise changes in the standard operating protocol for intravitreal injections at the Oxford Eye Hospital; **B** corresponding changes in the total number of intravitreal injections performed over time (line chart), together with the proportion of injections performed without a drape and speculum, or without a drape only (lid speculum alone) (bar chart); and **C** post-injection endophthalmitis rates per annum over the same period of time.

Our minimalist approach involves only the application of topical anaesthetic followed by povidone iodine drops in the conjunctival sac. Injectors are offered a single-use lid speculum and a sterile towel on which to place any instruments required, if they so wish. Many injectors opt not to use a lid speculum, instead using their fingers to gently retract the upper and/or lower lid, followed by IVI administration. The injection site 3.5–4.0 mm from the limbus is marked by the needle cap, which has a diameter of 4.0 mm, negating the use of a calliper (Fig. 2). Injections are performed in two adjacent outpatient clean rooms by a single injector.

**Fig. 2:**
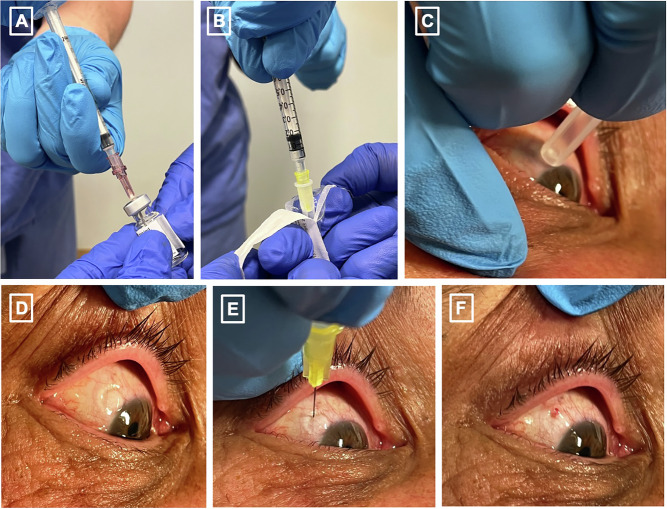
A minimalistic intravitreal injection technique that does not require the use of an intravitreal pack. **A** aseptically withdrawing the drug through a filter needle (an additional step which is unnecessary for pre-filled syringes), **B** attaching the syringe to a needle, **C** marking the injection site with the needle cap in place of callipers, **D** manual lid retraction demonstrating the mark, **E** manual lid retraction with delivery of intravitreal injection, **F** post-delivery of intravitreal injection. *Written informed consent for photography was obtained from the patient*.

We audited all consecutive IVIs administered in our outpatient injection suites between 1 January 2016 and 31 December 2024. We aimed to evaluate the environmental impact and safety (in terms of postoperative endophthalmitis rates as defined in [3]) of our updated IVI SOP. Pseudonymised data were extracted from the departmental electronic medical record system. To ensure data fidelity, diagnoses of post-IVI endophthalmitis were cross-referenced against local incident reporting logs.

Overall, 120,878 IVIs were performed in 12,044 eyes (9517 patients) over the study period. The number of IVIs increased between 2016 and 2024, apart from a slight dip during the Covid-19 pandemic in 2020. Annual numbers have risen from 8237 in 2016 to 21,828 in 2024, representing a 265% increase, highlighting a need for sustainable resource planning and service delivery models to accommodate the ever-growing demand.

Figure 1B shows local practice patterns following the change in SOP, with increasing adoption of the drape-free and speculum-free approach to injections over time. A standard intravitreal pack of the variety previously used at the Oxford Eye Hospital produces 143.2 g of waste (134.3 g of plastic, 1.1 g of metal, and 7.8 g of gauze) [4]. This means that applying our new SOP to the 21,828 IVIs performed over the most recent 12-month period in 2024 would have reduced waste generation by up to 3138 kg. Cost savings are estimated at £150,000–£180,000 per annum for the same volume of injections (for the intravitreal packs and gloves). Actual savings will vary depending on supplier pricing and commercial agreements.

There were 31 cases of post-injection endophthalmitis across the 9-year study period, giving an endophthalmitis rate of 0.026%, which was comparable to the incidence rate from a regional audit with standard conservative injection practices (0.024%) [3], and lower than that reported in meta-analyses of international studies (0.049-0.056%) [5, 6]. We excluded two cases with atypical presentations which were initially managed with a ‘tap-and-inject’ procedure in case of endophthalmitis but were attributed to medication-related inflammation. The endophthalmitis rates in the drape and lid speculum group (19/67316, 0.028%), lid speculum-only group (9/41509, 0.022%), and no drape or lid speculum group (3/12053, 0.025%) were not significantly different (*p* = 0.81, chi-squared test). There was no clear relationship between our stepwise changes in the injection SOP and the endophthalmitis rate, which demonstrated annual fluctuations independent of these changes (Fig. 1C).

We have presented a safe and evidence-based approach to minimising procedural waste for IVIs, which was performed in a stepwise manner, thus facilitating robust audit and feedback mechanisms. Our findings are corroborated by separate reports from the literature, where the omission of periocular cleaning, drapes, lid speculums [7–9], and/or switching to non-sterile gloves [10] either singly or in varying combinations was not associated with an increased risk of endophthalmitis [2].

Surveying patients about their experience would provide a more holistic perspective of these changes, though anecdotally, most patients prefer drape-free and speculum-free injections. This aligns with results from a previous survey, which outlined that drapes and speculums were the most uncomfortable part of the procedure outside of the injection itself [11]. We estimate from our personal experience that 3-5% of patients will continue to require lid speculums due to orbicularis oculi overaction, patient preference, and/or intravitreal implants such as Ozurdex® and Iluvien®. In addition, while most of our injectors welcomed the changes in the injection SOP, some injectors may still continue with lid speculums due to personal preference.

Overall, our minimalistic approach to performing IVIs offers a practical pathway towards sustainability without compromising patient safety, which is especially important in the context of the growing demand for IVIs. For a truly sustainable injection service, we must also consider broader aspects such as building energy, water consumption, patient and staff travel, and the environmental impact of drug and material manufacture and procurement [2]. Addressing these factors holistically will further optimise our carbon footprint and contribute to a more sustainable future.

## Data Availability

Data analysed during this study are included in this published article. More specific information is available from the corresponding author on reasonable request.
